# The Acids of Fruits

**Published:** 1895-05

**Authors:** G. W. Johnson


### THE ACIDS OF FRUITS.

The grateful acid of the rhubarb leaf arises from the malic acid and binoxalate of potash which it contains; the acidity of the lemon, orange, and other species of the genus *Citrus* is caused by the abundance of citric acid which their juice contains; that of the cherry, plum, apple, and pear from the malic acid in their pulp; that of gooseberries and currants, black, red, and white, from a mixture of malic and citric acids; that of the grape from a mixture of malic and tartaric acids; that of the mango from citric acid and a very fugitive essential oil; that of the tamarind from a mixture of citric, malic, and tartaric acids; the flavor of asparagus from aspartic acid, found also in the root of the marshmallow, and that of the cucumber from a peculiar poisonous ingredient called fungin, which is found in all fungi, and is the cause of the cucumber being offensive to some stomachs.

It will be observed that rhubarb is the only fruit which contains binoxalate of potash in conjunction with an acid. Beet-root owes its nutritious quality to about nine per cent. of sugar which it contains, and its flavor is a peculiar substance containing nitrogen mixed with pectic acid.

The carrot owes its fattening powers also to sugar, and its flavor to a peculiar fatty oil; the horse-radish derives its flavor and blistering power from a volatile acrid oil. The Jerusalem artichoke contains fourteen and a half per cent. of sugar and three per cent. of inulin (a variety of starch), besides gum and a peculiar substance to which its flavor is owing; and, lastly, garlic and the rest of the onion family derive their peculiar odor from a yellowish, volatile acrid oil, but they are nutritious from containing nearly half their weight of gummy and glutinous substances not yet clearly defined.—G. W. Johnson in the *Chemistry of the World.*

